# Integrating Norm Activation Model and Theory of Planned Behavior to Understand Sustainable Transport Behavior: Evidence from China

**DOI:** 10.3390/ijerph14121593

**Published:** 2017-12-18

**Authors:** Yuwei Liu, Hong Sheng, Norbert Mundorf, Colleen Redding, Yinjiao Ye

**Affiliations:** 1 Business School, Yangzhou University, Yangzhou 22509, China; liuyw@yzu.edu.cn (Y.L.); shenghong@yzu.edu.cn (H.S.); 2 Department of Communication Studies, Harrington School of Communication and Media, University of Rhode Island, Kingston, RI 02881, USA; nmundorf@uri.edu; 3 Department of Psychology, College of Health Sciences, University of Rhode Island, Kingston, RI 02881, USA; credding@uri.edu

**Keywords:** sustainable transport behavior, urban transport, car-transport reduction, intention survey, norm activation model

## Abstract

With increasing urbanization in China, many cities are facing serious environmental problems due to continuous and substantial increase in automobile transportation. It is becoming imperative to examine effective ways to reduce individual automobile use to facilitate sustainable transportation behavior. Empirical, theory-based research on sustainable transportation in China is limited. In this research, we propose an integrated model based on the norm activation model and the theory of planned behavior by combining normative and rational factors to predict individuals’ intention to reduce car use. Data from a survey of 600 car drivers in China’s three metropolitan areas was used to test the proposed model and hypotheses. Results showed that three variables, perceived norm of car-transport reduction, attitude towards reduction, and perceived behavior control over car-transport reduction, significantly affected the intention to reduce car-transport. Personal norms mediated the relationship between awareness of consequences of car-transport, ascription of responsibility of car-transport, perceived subjective norm for car-transport reduction, and intention to reduce car-transport. The results of this research not only contribute to theory development in the area of sustainable transportation behavior, but also provide a theoretical frame of reference for relevant policy-makers in urban transport management.

## 1. Introduction

With the acceleration of urbanization and increased per capital income of urban residents, China has rapidly reached an era of motorized urban transportation [1], and private car ownership in China reached 172 million by 2016 [2]. However, this rapid growth of private car ownership has negatively affected the environment and produces traffic congestion and accidents, air pollution, and excessive noise; it has also severely depleted fossil fuels [3]. Statistics show that using public transit services instead of private cars could reduce gasoline consumption by approximately 83%; and substituting only 200 km per year traveled by each private car in China with public transit or other sustainable transportation modes could save more than 2 billion liters of gasoline, and carbon dioxide emissions could be reduced by 4.56 million tons. Reducing single occupancy vehicles (SOVs), therefore, would not only have an economic advantage, but also greatly reduce the carbon footprint and preserve the planet. Moreover, this reduction has tremendous benefits to public health. High levels of transport-related air pollutant concentration are linked to increased risk for cardiovascular and respiratory disease, cancer, birth defects, and higher death rates [4]. An estimated 3.7 million premature deaths are attributable to outdoor air pollution according to World Health Organization (WHO) data from 2012 [4]. Transport-related noise stress also poses a significant risk for public health. Adopting sustainable transportation modes such as walking and cycling can not only reduce air and noise pollution, but also promote an active and healthy lifestyle. 

It is then important to examine various effective ways to reduce private automobile use and encourage sustainable transport behavior. Previous research has used mainly the Theory of Planned Behavior (TPB) to predict the intention to reduce car use and sustainable transportation behavior; some studies have investigated other factors, such as personal norm and car use habit, to explain the intention or behavior [5,6,7,8,9,10,11,12,13,14,15,16,17,18]. In particular, Heath and Gifford [11] tested the predictive utility of TPB in a sample of Canadian university students. Lois and colleagues [16] incorporated social identity into TPB to predict cycle commuting. Gardner and Abraham’s meta-analysis [7] found a general support for TPB, but a weak relationship between pro-environment cognition and private car use. Lanzini and Khan’s meta-analysis [13] supported TPB and found environmental concern was related to the intention to choose a greener transport mode rather than actual transport choices. Other studies found that personal norm is a significant predictor of willingness to reduce car use and travel behavior [17,18]. The study by Bamberg et al. [6] incorporated TPB, the Norm Activation Model (NAM) and the Theory of Interpersonal Behavior, but personal norm was not a significant predictor of intention to reduce car use in the integrated model; and the study surveyed a group of students in 1997. Our study follows Bamberg et al.’s study [6] and integrates TPB and NAM to examine intention to reduce car transport; it also advances this field through a survey of the general population in China, rather than students. Our study is also a response to the fact that very limited research on this topic has been done in China [3,19,20]. Li and Jones’ study [3] analyzed the impacts of restricting vehicle ownership by the Beijing municipal government on greenhouse gas emission. Geng et al.’s study [19] examined how an informational campaign promoted the adoption of green transport modes. Mao et al.’s study [20] examined how travel modes relate to travel satisfaction.

The integration of the two models in our study is based on our observation that in most cases, car-transport reduction is a function of a mixture of pro-social concerns [17,18,19] and self-interest [19]. TPB posits that one important determinant of a person’s behavior is perceived subjective norm (i.e., how people important to the person think of the behavior). NAM posits individuals’ behavior (e.g., reduce private car use) is predicted by personal norm (i.e., internal standards concerning a particular behavior) rather than social norm that reflects externally imposed rules [21]. Therefore, the two theoretical models are useful in our investigation. 

Reducing automobile use is more challenging than promoting recycling, domestic energy conservation, and other pro-environmental behaviors, because the latter are relatively easier to implement [22,23]. Transport Demand Management (TDM) describes a variety of actions used to modify or reduce demand on transportation services and facilities [24]. Coercive measures, such as government-imposed fuel taxes, road charges and vehicle access restrictions, although effective sometimes, are costly in implementation and also face great opposition from car drivers. Consequently, it is important to explore more cost-effective and sustainable measures geared towards attitude and behavior change to promote voluntary reduction of automobile use in China, in particular SOV transportation. To this end, TPB and NAM that deal with personal awareness, attitudes and beliefs provide insight, and the following gives a detailed review of relevant theories and studies in this area. 

### 1.1. Sustainable Transport System and Car Transport Reduction 

The European Conference of Ministers of Transport states that a sustainable transportation system: (a) “permits the basic access needs of individuals and societies to be met safely and in a way consistent with human and environmental health, and with equity within and between generations”; (b) “is inexpensive, operates efficiently, offers choices of transport mode, and supports an energetic economy”; and (c) “restricts emissions and waste within the planet’s ability to dispose of them, minimizes consumption of non-renewable resources, limits consumption of renewable ones to the sustainable yield level, reuses and recycles its components, and minimizes the use of land and the production of noise” [25]. 

Improving automobile energy efficiency and thus reducing greenhouse gas emissions and resource use can be a way to achieve a sustainable transport system [26]. Energy efficiency may be improved through technological advances, such as efficient engines, hybrid fuel systems, and fuel cell technology, thus mitigating some adverse impacts of car-transportation on the environment. However, technological advancement often comes with increased car ownership and automobile use, which offsets its positive effect on reduced energy consumption [27]. Consequently, energy efficiency resulting from technological advancement itself is not sufficient to promote a sustainable transportation system; introducing other measures to achieve a sustainable transportation system becomes necessary.

#### Transport Demand Management 

TDM measures can be classified into two categories: push measures that discourage car use and pull measures that encourage alternative transportation modes [28]. Vlek and Michon [29] also suggest the following TDM strategies to implement car-transport reduction policies (ordered from more to less coercive: (1) physical change by providing alternative transportation (2) laws and regulations (3) economic incentives (4) information, education, and prompts (5) socialization and social modeling targeted at changing social norms and (6) institutional and organizational changes. 

Some TDM measures aim to change existing transportation options (e.g., increasing road tolls to reduce traffic congestion) and others target at car drivers’ psychological characteristics without increasing transportation costs [30]. Research has shown that the latter ones, such as increasing public awareness of environmental problems due to excessive car use and personal norms with regard to car-transport, are more effective in changing individuals’ transportation habits in favor of sustainable transportation behaviors because these measures give people the freedom to choose their own transportation modes [5,28,31,32].

China recently has increased its efforts to reduce automobile use amid the serious environmental pollutions that it is facing. Government-imposed regulations, such as pricing mechanisms (e.g., fuel taxes, vehicle import, purchase, registration taxes, direct road charges, parking fees) and vehicle access restrictions (e.g., limits on car use based on certain criteria), although effective to some extent, have unintended consequences (e.g., drivers purchase additional cars to circumvent the restrictions). Regulatory approaches also face opposition from the auto industry and vehicle owners [6], and there is no guarantee that their benefits will exceed implementation and maintenance costs. In contrast, informational campaigns have been used to provide relatively low-cost information about private-car-use social norms, risks of one’s own transport choices, and risk reduction strategies. Various promotional strategies have been used, such as social modeling, social support, appeal to commuters’ conscience, and appeal to altruism towards others and future generations. Although informational campaigns are effective in favor of sustainable transport, they are generally limited, with varying degrees of success [33] because private motorized vehicles have been long associated with individuality, hedonism, pleasure, comfort, speed, convenience, freedom, power, and superiority [34]. Therefore, regulatory approaches must be combined with transport policies and promotional measures in order to be successful. Promotional campaigns can improve the public’s acceptance of sustainable transport systems and make coercive TDM measures more acceptable and politically feasible. 

### 1.2. Theoretical Foundations

The Norm Activation Model (NAM) explains altruistic and environmentally friendly behavior and can be used as a theoretical basis for car-transport reduction research, because the personal interests of a driver need be given up for the sake of the environment, and thus are regarded as a similar form of pro-social behavior or altruism [35,36]. According to NAM, an individual’s pro-environmental behavior is determined by the degree of his/her personal responsibility for such behavior, which is reflected in personal norm (PN). Depending on how consistent an individual’s behavior is with his or her personal norm, the person may develop a sense of pride or guilt. Furthermore, NAM assumes that the process of norm activation is determined by two factors, awareness of consequences (AC) and ascription of responsibility (AR). In other words, if an individual is aware of problems caused by certain behaviors, this awareness is followed by the consideration of his or her own contribution to those problems and whether or not he or she can help solve such problems. Those variables of NAM have been used in the area of sustainable transport behaviors, such as interpreting the change in transport pattern [6,10,11,15,17,37,38,39,40,41] and acceptance of TDM measures [42].

Different from NAM—which is confined to ethical behavior, the Theory of Planned Behavior (TPB) has been used to analyze a variety of deliberate behaviors, and is considered one of the most influential theories in social psychology. TPB assumes that an individual’s intention is a direct determinant of his or her behavior, and one’s intention is the function of attitudes, subjective norm (SN) and perceived behavioral control (PBC) [43]. Applying TPB to car-transport reduction, first, attitudes describe the degree of personally favorable or unfavorable evaluation of car-transport reduction. Secondly, subjective norm refers to the perceived social pressure for execution or non-execution of car-transport reduction. Finally, PBC addresses one’s perception of efficacy and difficulty of carrying out car-transport reduction.

TPB has been widely used in the area of transportation and environmental psychology [44,45]. For instance, Heath & Gifford [11] found a significant positive correlation between self-reported bus use and the intention to take a bus, which in turn is positively correlated with attitudes towards bus use, subjective norm and PBC of bus use among college students in a Canadian university. In addition, a study on the acceptance of driving restriction policies in Tianjin, China found that car-owners had more negative attitudes towards driving restriction policies and public transport than non-owners, and there was a positive correlation between attitudes towards public transport and commuting time [46]. Similarly, Donald et al. [47] found that there were positive correlations between attitudes, PBC, and car-transport in their study. 

NAM and TPB are two empirically supported but distinctive frameworks to explain human behaviors. However, one might question whether it is necessary to regard car-transport reduction as either completely motivated by pro-social or self-interested motives. In most cases car-transport reduction can be a function of a mixture of self-interest and pro-social concerns. Therefore, these two theories, if combined, can offer complementary perspectives to explain car transport reduction behavior. Specifically, NAM mainly focuses on altruism assumptions that the personal norm is the determinant of pro-social behavior [36], and that pro-environmental behavior, according to TPB, is the outcome of rational analysis of personal costs and benefits. Research on pro-environmental behavior in light of either NAM or TPB has offered insights into the understanding of car-transport reduction behavior. For instance, previous research based on NAM or personal norm has focused on the predictive ability of moral consideration [5,6,11,17] whereas studies based on TPB are concerned with the predictive ability of beliefs about private car use, such as cost, convenience, travel time, comfort, and flexibility) [13,14,16,18]. There is, however, still a lack of understanding on the antecedents of intention to reduce car-transport in China. The objectives of this study are twofold: to propose a research model integrating NAM and TPB, and to empirically test the integrated model to explain the influence of normative and rational factors on the intention to reduce car-transport in China. 

### 1.3. Research Model and Hypothesis Development

Research on pro-environmental behaviors based on NAM found that the awareness of environmental consequences and the attributed responsibility of mitigating those environmental consequences are important antecedents to pro-environmental behaviors. In general, if a person is conscious that his or her own behavior brings negative consequences on others and the natural environment (i.e., AC), (s)he may feel responsible for these negative effects, and therefore, may believe that his or her environmentally responsible behavior helps alleviate environmental problems (i.e., AR) and consequently activate his or her personal norm [9,11,48,49]. In other words, individual’s problem awareness is the first step towards responsible actions, and the degree to which he or she considers solving the problem through his or her own behavior in turn activates his/her personal norm and determines the degree of activation [38,40]. Thus, it is expected that the awareness of consequences of car-transport (AC) and the ascription of responsibility for sustainable transport behavior (AR) will influence the personal norm of car-transport reduction (PN), which in turn has an impact on the intention to reduce car-transport. Therefore, a set of hypotheses are developed as follows and we have tested these hypotheses in a sample of Chinese car drivers:

**Hypothesis** **1a** **(H1a).** An individual’s awareness of car-transport’s consequences (AC) has a positive impact on personal norm of car-transport reduction (PN).

**Hypothesis** **1b** **(H1b).** Personal norm of car-transport reduction (PN) mediates the relationship between awareness of car-transport’s consequences and intention to reduce car-transport.

**Hypothesis** **2a** **(H2a).** An individual’s ascription of responsibility for car-transport reduction (AR) has a positive impact on his or her personal norm of car-transport reduction (PN).

**Hypothesis** **2b** **(H2b).** Personal norm of car-transport reduction (PN) mediates the relationship between ascription of responsibility for car-transport reduction and intention to reduce car-transport.

**Hypothesis** **3** **(H3).** Personal norm of car-transport reduction (PN) has a positive impact on intention to reduce car-transport.

TPB suggests that subjective norm (the perceived social pressure to perform or not to perform the behavior in question) and normative belief (belief about whether each referent approves or disapproves of the behavior) are considered when one has to make choices among a range of behaviors. In addition, both necessary resources and potential barriers that either facilitate or constrain the occurrence of the behavior are also considered, such as whether pro-environmental transportation modes are convenient and practical [43]. In summary, attitude, perceived norm, and control beliefs influence individuals’ intention to perform the behavior and TPB posits that a behavior with more favorable outcomes will be preferred than its counterpart [11,14,16,45]. Thus we propose the following hypotheses based on TPB in the context of transportation behavior in China:

**Hypothesis** **4** **(H4).** Attitude towards car-transport reduction has a positive impact on the intention to reduce car-transport.

**Hypothesis** **5** **(H5).** Subjective norm of car-transport reduction (SN) has a positive impact on the personal norm of car-transport reduction.

**Hypothesis** **6** **(H6).** PBC over car-transport reduction has a positive impact on the intention to reduce car-transport.

According to NAM, subjective or social norm reflects the perceived expectations of important reference persons; and has an impact on the behavior, because people are afraid of social sanctions if they do not obey the subjective or social norm. Personal norm, nonetheless, pertains to an individual’s belief that acting in a particular way is personally approved or not approved. Therefore, the key feature of personal norms is internalization, that is, an individual’s willingness to act in concert with her/his own personal norm is not based on his/her fear of social sanctions, but the anticipation of negative personal feelings, such as remorse or guilt after having broken her/his personal norm.

In this research, we propose an integrated framework by establishing relationships among variables from TPB and NAM. We hypothesize that the subjective norm takes precedent over the personal norm, because the importance of car-transport reduction in society is recognized by subjective norm which may then be internalized as personal norm by the individuals who are subject to the pressures from important referees [50]. In other words, it is anticipated that the popularity of beliefs for car-transport reduction in society will lead individuals to take responsibility for reducing car-transport. The internalization of social norms is a social construction process influenced by the interaction or dialogue with significant reference persons who interpret and frame rules within a personal context [51]. This is supported in many studies that the opinion “climate” of the encompassing social context strongly influences one’s beliefs about societal problems [52,53]. Research in the pro-environmental behavior domain also provides empirical support for a subjective norm—personal norm connection [50,54,55,56,57]. Specifically, individuals’ perception that reducing car-transport is socially desirable may influence their feeling of personal obligation and intention to reduce car-use. Accordingly, the following hypotheses are put forward:

**Hypothesis** **7a** **(H7a).** Subjective norm of car-transport reduction (SN) has a positive impact on the intention to reduce car-transport.

**Hypothesis** **7b** **(H7b).** Personal norm of car-transport reduction (PN) mediates the relationship between the subjective norm of car-transport reduction and the intention to reduce car-transport.

**Figure 1:**
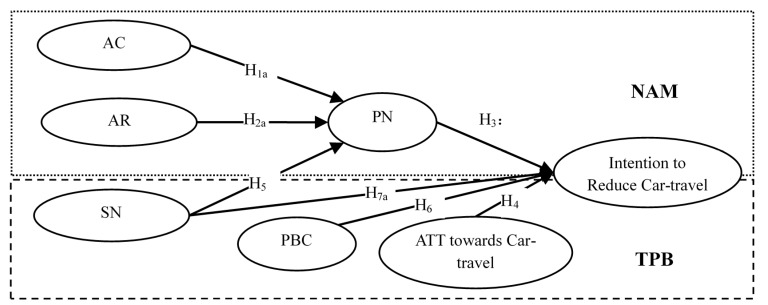
Conceptual Framework. Note: AC = Awareness of consequence of car-travel; AR = Ascription of responsibility for car-travel reduction; PN = Personal norm of car-travel reduction; SN = Subjective norm of car-travel reduction; PBC = PBC over car-travel reduction.

In summary, a research framework on antecedents of car-transport reduction is formed as shown in Figure 1. Hypotheses reflecting the mediating effects of personal norm including H1b, H2b and H7b are not displayed in Figure 1.

## 2. Materials and Methods 

In order to ensure reliability and validity of measurement scales, established scales from key literature discussed in the earlier sections were adapted to measure the key variables of this research. Existing scales were adjusted after in-depth interviews about the items of measurement. The measurement scale was finalized based on the results of a pre-survey with 30 participants. In this research, all the constructs were measured on 7-point scales.

### 2.1. Measures

#### 2.1.1. Measures of NAM Constructs: AC, AR and PN

Measures of NAM constructs were identified from the previous research on car-transport reduction in light of NAM [20,21,22,23,24,25,26,27,28,29]. 

AC. Four items were summed to measure the respondent’s opinion on the extent to which negative consequences of car-transport are regarded as environmental problems: ‘Increasing car-transport leads to gradual depletion of fossil fuel’; ‘Car-transport is one of the important causes of traffic jam and related traffic accidents’; ‘Car-transport causes traffic noise and exhaust emission, lowering the quality of city life’; and ‘Increasing car traffic is a very serious problem for me and my families’. Higher ratings reflect the respondent’s stronger awareness of environmental problems caused by car-transport.

AR. The following two items were summed to measure the degree to which the respondents ascribe the problems caused by car-transport to their responsibilities: ‘As a driver, I bear joint liability for gradual depletion of fossil fuel caused by car-transport’; ‘Car transport is one of the causes of global warming for which I am jointly and severely liable as a driver’. Higher scores indicated higher ascribed personal responsibility.

PN. The following three items were summed to measure the respondent’s personal norm of car-transport reduction: ‘If I often use other modes of transport rather than car, I would be a more responsible person’; ‘When forming transport options, I feel I’m obliged to consider the environmental consequences of car-transport’; ‘I feel that I’m morally obliged to minimize car usage regardless of others’ behavior’. Higher scores indicated higher personal norm. 

#### 2.1.2. Measures of TPB Constructs: Attitude, SN and PBC, Intention

Three constructs of TPB were identified based on previously described research on car-transport, especially drawing on Ajzen’s research on measurement of TPB constructs [43,58].

Attitude towards car-transport reduction. Four items were summed to measure the respondent’s attitude towards car-transport reduction with a 7-point Semantic Differential scale. Participants were asked to rate their attitude toward car-transport reduction: ‘Harmful/beneficial’, ‘Disgusting/pleasant’, ‘Bad/good’ and ‘Unworthy/valuable’. Higher scores meant a more favorable attitude toward car-transport reduction. 

SN. Three 7-point scale items were established: ‘I’m supported by most of the people important to me to reduce car-transport’, ‘Most of the people important to me think that I should reduce car-transport’; and ‘The government’s policy on the priority to the development of public transport supports me to reduce car-transport’. These three items were designed to measure the respondents’ beliefs in expectation from important others (i.e., relatives, friends, colleagues, transport management agencies) to reduce car-transport. Higher scores on the sum of these items indicate that the respondents endorses subjective norm of car-transport reduction to a greater extent.

PBC. Three 7-point scale items were used to measure efficacy and difficulty of car-transport reduction perceived by the respondents: ‘Whether my car-transport can be reduced or not completely depends on me’; ‘If I want, I can easily reduce my car-transport’; ‘I have enough time and energy to take control over my car-transport reduction’. Higher scores reflected a higher level of perceived behavior control regarding car-transport reduction. 

Intention to reduce car-transport. Four 7-point scale items adapted from Jakovcevic and Steg [40] were used to measure the respondent’s intention to reduce car-transport in four major daily activities, i.e., ‘In the following activities (work, shopping, social activities and recreation), your possibility of reducing car-transport and switching to public transport, walking or cycling for transport is’ (1 means ‘definitely not’ and 7 means ‘definitely’). Higher scores reflected a stronger inclination to reduce car-transport. 

### 2.2. Procedures and Respondents

In August 2014, we surveyed car drivers in three major metropolises in China, including Beijing, Shanghai, and Guangzhou, with the help of a well-known market research company, which had access to a pool of over 1.2 million car-drivers. This sampling technique ensures broad geographical distribution of respondents from major cities in China. Among the 1.2 million car-drivers, 11.7% were from Beijing, 11.1% were from Shanghai, and 9.4% were from Guangzhou. The survey was sent to 9000 people, and 600 participants responded to our survey. The response rate was about 6.7%. Table 1 demonstrates the participants’ demographics.

### 2.3. Analytical Methods 

Structural equation modeling (SEM) was adopted in this research to simultaneously test multiple relationships in the research model as shown in Figure 1. Two major methods are often used for SEM analysis in existing research: one method is based on component analysis (such as partial least square, namely PLS) and the other on covariance matrix (such as LISREL and Amos) [59]. PLS structural equation modeling technology was selected for this research to test the hypotheses for the following reasons. Firstly, constructs of multiple items can be analyzed by PLS, which is widely used in the field of behavioral research. Secondly, PLS doesn’t need a priori requirement for the items that must be subject to an independent and multivariate normal distribution. Compared to covariance based SEM methods, PLS does not require larger sample size, and better prediction effect may be achieved through limited sample sizes [59,60]. Thirdly, the PLS method is suitable for testing complex relationships without requiring strong theoretical justification [61]. Fourth, moderating and mediating effects among variables can also be detected by PLS method even with measurement errors. Since most of our survey data was not normally distributed and we wanted to test relationships among a number of variables, the choice of PLS as analysis method seemed reasonable. SmartPLS 2.0 M3 developed by Ringle et al. [62] at University of Hamburg, Germany was used in this research. We first evaluated the reliability and validity of measurement model and then tested the research hypotheses. The mediation relationships were tested using SPSS’s PROCESS macro. 

## 3. Results

### 3.1. Reliability and Validity of Measurement Model 

In this research, validity and reliability analysis was conducted for all constructs. Specifically, internal consistency and composite reliability were tested. As shown in Table 2, both Cronbach’s αs and composite reliability indices of all the measures had values greater than 0.7, indicating that the measures were reliable. Furthermore, scale validity was verified by convergent validity and discriminant validity of the constructs. Specifically, confirmatory factor analysis was used to verify convergent validity. Model fit indices showed that: e^2^ (174) = 237.53, a^2/*df*^ = 1.36 (less than 5), RMSEA = 0.025 (less than 0.05), CFI = 0.97, NNFI = 0.97, IFI = 0.99 (greater than 0.9). Also shown in Table 2, Average Variance Extracted (AVE) of all the constructs were greater than 0.5 [63]. Thus, all the indicators above showed high convergent construct validity. Discriminant validity was verified by the square root of AVE of each construct, and the correlation coefficient between this construct and other constructs. Square root of AVE of each construct was indicated by values on the diagonal in Table 3, and correlation coefficients between constructs were indicated by values on the non-diagonals in the table [63]. Square roots of AVEs of constructs on the diagonal were greater than correlation coefficients on the non-diagonals between constructs, indicating good discriminant validity among constructs. In summary, this measurement model demonstrated high reliability and validity. 

### 3.2. Test of Common Method Biases

As a covariation property derived from measurement methods rather than research constructs, common method biases (CMB), a systematic error variance shared among variables measured by the same method and/or source, can produce misleading results and conclusions. It is necessary to test CMB to detect possible false relationships among the constructs. There are two methods to test CMB. The first one is Harman’s single factor testing method, which involves an unrotated exploratory factor analysis of the constructs’ measures [64] to determine the number of factors that are necessary to account for the variance in the variables. If a single or general factor explains more than 50% of the covariance among the constructs’ measures then it can be concluded that a substantial amount of common method variance is present. Using SPSS 22.0 (Chicago, IL, USA), we found that the first factor explained 42% of the constructs’ variance, indicating CMB was at an acceptable level. The second method to test CMB is to test correlation among constructs and high CMB is present if correlation coefficients among constructs are greater than 0.9. As shown from Table 3, correlation coefficients among constructs are between 0.335 and 0.648 and therefore, the CMB was acceptable. Based on the results of the two CMB test methods, we can conclude that our data was not affected by common method biases.

### 3.3. Structural Model and Mediation Tests

**Figure 2:**
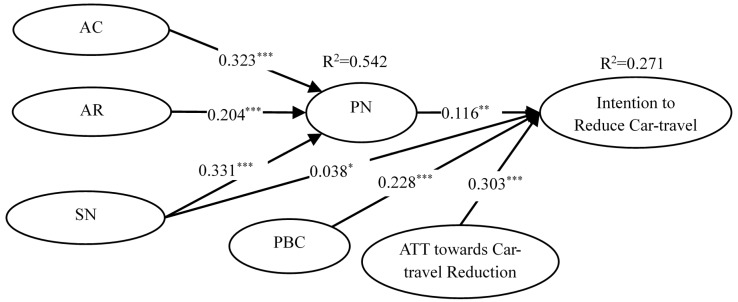
Standardized Path Coefficients of the Model. Notes: AC = Awareness of consequence of car-travel; AR = Ascription of responsibility for car-travel reduction; PN= Personal norm of car-travel reduction; SN = Subjective norm of car-travel reduction; PBC = PBC over car-travel reduction. * *p* < 0.05; ** *p* < 0.01; *** *p* < 0.001.

SmartPLS was used to test the structural model. We first used the PLS algorithm from SmartPLS to calculate the path coefficients of the structural equation model. Different from LISREL and AMOS, PLS is used to determine the path coefficients of the model and to measure the predictive power of structural equation model with R^2^. When R^2^ ≤ 0.02 a weak path relation is indicated; a moderate path relation is indicated with 0.02 < R^2^ ≤ 0.13; a strong path relationship with 0.13 < R^2^ ≤ 0.26 [49]. In addition, the statistical significance of each path coefficient was tested through Bootstrapping method provided by PLS. As shown in Figure 2, PN, SN, PBC, and attitude combined explained 27% of the variance of intention to reduce car-transport, R^2^ = 0.27; and AC, AR, and SN combined explained 54% of the variance in PN, R^2^ = 0.54.

The test results are shown in Table 4. PN was an outcome variable of AC (β = 0.32, *t* = 7.66, *p* < 0.001), AR (β = 0.20, *t* = 5.39, *p* < 0.001), SN (β = 0.33, *t* = 8.12, *p* < 0.001), and a predictive variable of intention to reduce car-transport (β = 0.12, *t* = 2.80, *p* < 0.01); therefore H1a, H2a, H5 and H3 were supported, respectively. Path coefficients between attitude towards car-transport reduction and car-transport reduction intention (β = 0.30, *t* = 6.995) and between PBC and intention to car-transport reduction (β = 0.23, *t* = 5.72) were statistically significant at the level of *p* < 0.001, thus H4 and H6 were supported. Similarly, the path coefficient between SN and intention to reduce car-transport (β = 0.04, *t* = 2.57) was significant at the level of *p* < 0.05, thus H7a was also supported.

As shown in Figure 2, the paths from AC to PN and from PN to intention were significant, and so were the paths from AR to PN and from PN to intention. The paths from SN to PN and from PN to intention were also significant. To test the statistical significance of the indirect effect of AC, AR and SN on intention to reduce car-transport, we conducted mediation analyses using SPSS PROCESS macro. Results based on 5000 bootstrapped samples showed that the standardized indirect effect of AC on intention to reduce car-transport mediated by PN was β = 0.11 (S.E. = 0.04) with a 95% confidence interval [CI] between 0.04 and 0.19 (see Table 4). Therefore, the mediation effect was statistically significant, and AC was associated with approximately 0.11 higher scores of intention to reduce car-transport as mediated by PN. Similar bootstrapping procedures were conducted to test the mediation of PN between AR and intention to reduce car-transport, and results showed that the indirect effect was statistically significant, β = 0.13 (S.E. = 0.04), 95% CI [0.06, 0.22]. The indirect effect for the mediation of PN between SN and intention to reduce car-transport was β = 0.08 (S.E. = 0.04), 95% CI [0.002, 0.38], therefore the mediation effect was significant. Therefore, PN was a significant mediator in each of the three relationships, and the three mediation hypotheses H1b, H2b, and H7b were supported. 

With regard to control variables, intention to reduce car-transport was significantly affected by drivers’ educational level (β = 0.06, *t* = 2.19, *p* < 0.05) and years of driving, (β = −0.05, *t* = 2.04, *p* < 0.05), indicating that more educated drivers had a higher level of intention to reduce their car-transport; in contrast drivers with more years of driving experience were more reluctant to reduce their car-transport than their counterparts. Other control variables had no significant associations with intention to reduce car-transport.

In summary, the results indicated that compared to attitude towards car-transport reduction and PBC, personal norm had a weaker relationship with intention to reduce car-transport, suggesting that NAM had less explanatory power on a relatively difficult pro-environmental behavior (such as car-transport reduction). It may be concluded that TPB has a stronger predictive power than NAM in predicting eco-friendly behaviors. The relationships between all antecedent and consequence variables have been supported by our data; additionally the hypothesis of personal norm as a mediating variable was also supported. All these results indicated that an integrated model based on NAM and TPB about intention to reduce car-transport was supported by our survey data.

## 4. Discussion

This research examined the antecedents of car-transport reduction in China by integrating two theories, namely NAM and TPB. The research findings suggested that two variables of NAM, i.e., awareness of consequence and ascription of responsibility, had significant positive impacts on personal norm, which in turn had a significant positive impact on intention to reduce car-transport. That is, personal norm acted as a significant mediating variable between antecedent and consequent ones. In addition, two variables of TPB, namely attitude towards car-transport reduction and perceived behavioral control, also had significant positive impacts on intention to reduce car-transport, while subjective norm was internalized into personal norm which mediated the impact of subjective norm on intention to reduce car-transport. These findings make a significant contribution to the understanding of sustainable transport behavior in China [3,19,20]. The current study is one of the first to empirically test car-use-reduction intention and surveyed car drivers in three metropolises in China. The present study has also contributed to the literature theoretically by integrating TPB and NAM and demonstrating the significance of personal norm, along with the TPB variables, in predicting the intention to reduce car use. Previous studies have mainly relied on TPB to examine car-use intention and behavior, and our study tested an integrated model based on TPB and NAM in China.

Another interesting result is that compared to the variables in TPB (i.e., social norm, attitude towards car-transport reduction and perceived behavioral control), personal norm alone had a weaker relationship with intention to reduce car-transport, suggesting that NAM had less explanatory power than TPB on car-transport reduction, a behavior relatively difficult to be implemented. This finding is consistent with the Bamberg et al.’s study [6] that integrated TPB and NAM and found no significant relationship between personal norm and the intention to reduce car-use, when factors from TPB were included to predict the intention. Similarly, Gardner and Abraham (2008)’s meta-analysis [7] demonstrated a general support of TPB and a weak relationship between pro-environment cognition and private car use. Personal norm is found to be significant in predicting the acceptance of specific TDM measures such as increased tax on fuel and improved public transport [5], but support for these measures does not automatically translate into the intention to reduce car use or actual reduction in private car use. 

Research on adopting pro-environment behaviors based on NAM or including personal norm as a predictor finds that personal norm is a strong predictor of electricity saving behavior (β = 0.35) [65]. Chan and Bishop [66] found a significant relationship between personal norm and recycling intention (β = 0.33), and ultimately actual recycling. As can be seen in these studies, personal norm is a medium-to-strong predictor of pro-environment behaviors such as recycling and energy saving, whereas personal norm is a weaker predictor of car-transport reduction. It is plausible that pro-environment behaviors such as energy-saving and recycling are different from driving an automobile, which is often associated with individuality, hedonism, freedom, pleasure, comfort, speed, convenience, superiority and power [34]. These other environmental behaviors are more easily implemented than car-transport reduction. However, we still maintain the relative usefulness of personal norm in predicting intention to reduce car-transport and the actual behavior, because of its significant prediction of the intention and behavior [7,15,17]. Moreover, personal norm mediates the effect of social norm, a TPB variable, on intention to reduce car use. Therefore, an integrated model based on NAM and TPB can shed light on the examination of intention to reduce car-transport and sustainable transport behavior.

Many cities in China are facing increasingly serious problems of resource constraints and suffering from environmental pollution due to a substantial increase in car transport. Based on NAM and TPB, we proposed an integrated model that demonstrates both social norms and personal rational motives (e.g., whether pro-environmental behaviors are beneficial to individuals and effective in protecting environment) are important determinants of intention to reduce car use. These results suggest that efforts to promote sustainable travel behavior may fall short without accounting for the psychological variables, and has some important implications for the effectiveness and efficiency of TDM in China. 

First, because both awareness of consequences and ascription of responsibility influence personal norm, which subsequently influences intention to reduce car transport, soft TDM policies such as education and awareness campaigns should be adopted to promote the awareness of negative environmental consequences of car-transport, such as fossil fuel consumption, traffic jam and accidents, noise, toxic greenhouse gas emissions, and global warming. Besides targeting car users, the government should also impose regulations that require automobile commercials to include a disclaimer about the environmental consequences of private mobile use. Moreover, education can also enable individuals to take more personal responsibility for protecting natural environment and to internalize social norm of reducing car-transport. The internalization process in which an individual accepts a set of norms and values established by others through socialization is important in the formation of car-transport-reduction intention, as our results show. Second, because our study demonstrates that personal attitude and perceived behavioral control significantly predicted the intention to reduce car transport, educational campaigns should also provide people with adequate information on alternative transportation modes and guide them to form positive attitudes towards alternative transport modes. These awareness campaigns may not be as effective as one would expect, because the actual behavior, car-transport reduction, is harder to change than other pro-environmental behaviors as previously discussed. Therefore, awareness campaigns should also be combined with regulatory measures (e.g., support for non-motorized modes, investment in public transportation, pricing mechanisms that discourage private car use, and vehicle access restrictions) to promote sustainable urban transport and address some urban environmental problems. In other words, TDM policies aimed at increasing bottom-up voluntary change of car-transport behavior, combined with top-down compulsory actions, are critical to promote sustainable transport behavior in China, and the combination of measures can lead to impacts greater than the sum of their individual parts. Reducing car transport through a combination of education campaigns and regulatory measures will greatly improve China’s environmental conditions by drastically reducing greenhouse gas emissions and air pollutants. On-road vehicles are a primary generator of PM_2.5_ (particulate matter with diameters of 2.5 micrometers or less) [67] and these tiny pollutants can embed in lungs and enter the bloodstream, leading to heart and respiratory problems, according to the WHO [4]. The reduction of greenhouse gas emissions and air pollutants because of people choosing pro-environmental transportation modes, will benefit Chinese people in many ways, as the WHO puts it: “Shifting from private motorized transport to rapid transit/public transport, such as rail, metro and bus, is associated with a wide range of potential health and climate benefits, including: lower urban air pollution concentrations, lower rates of traffic injury risk, less noise stress and improved equity of access for people without cars” [68].

In spite of some important theoretical and practical implications, limitations of this research must be noted. Although more random sampling would have been stronger, our survey respondents were from three metropolitan cities in China, Beijing, Shanghai and Guangzhou, all with very intensive car usage. Furthermore, since this survey was conducted online, the population of drivers in China may not be fully represented by this sample, since not all drivers can be reached online. Future research may extend this study by collecting data from more representative samples or data could be collected from areas with lower car-transport density to increase variability. Finally, this was a cross-sectional survey, and future studies should replicate and extend these results longitudinally. Future research could also examine the relationship between intention to reduce car-transport and actual reduction behavior. Furthermore, more research needs to be done to understand stages of transport behavior change and barriers to such behavior change (such as transport habits). We need to better understand driver characteristics, perhaps by segmenting drivers by motivational variables of transport behavior using cluster analysis. Last but not least, field studies on some TDM measures are necessary to evaluate the effectiveness of different TDM measures to promote sustainable transport.

## 5. Conclusions

In this research, we propose an integrated model based on the Norm Activation Model and the Theory of Planned Behavior by combining normative and rational factors to predict the intention to reduce car-transport. Data was collected by surveying 600 car drivers across China’s three metropolises to test the proposed model and hypotheses. The results show that three variables, personal norm of car-transport reduction, attitude towards car-transport reduction, and perceived behavior control over car-transport reduction, significantly affected the intention to reduce car-transport, and that personal norm mediated the impacts of awareness of consequences of car-transport, ascription of responsibility of car-transport, subjective norm of car-transport reduction on intention to reduce car-transport. The results of this research not only contribute to theory building in the area of sustainable transport behavior, but also provide a theoretical reference for relevant policy-makers of urban transport management.

## Figures and Tables

**Table 1:** Participants’ Demographics.

Variable	Percentage
Age	
<25	5%
25–29	25%
30–34	30%
35–39	25%
40–49	10%
50	5%
Education	
Less than high school	0.2%
High school or vocational education	2.5%
Associate degree	17.2%
Bachelor’s degree	72.2%
Master’s degree or higher	8.0%
Income	
Less than RMB ¥100,000	4.5%
RMB ¥100,000–150,000	20.3%
RMB ¥200,000–300,000	35.2%
RMB ¥300,000–500,000	12.7%
RMB ¥500,000 or more	2.0%

**Table 2:** Results of Reliability Tests.

Variable	AVE	Composite Reliability	Cronbach’s α
AC	0.676	0.893	0.839
AR	0.795	0.886	0.743
PBC	0.703	0.876	0.788
PN	0.758	0.904	0.840
SN	0.668	0.858	0.749
Attitude towards car-transport reduction	0.704	0.904	0.859
Intention to reduce car-transport	0.586	0.849	0.762

Notes: AVE = Average Variance Extracted. AC = Awareness of consequence of car-transport; AR = Ascription of responsibility of car-transport reduction; PN = Personal norm of car-transport reduction; SN = Subjective norm of car-transport reduction; PBC = Perceived behavioral control over car-transport reduction.

**Table 3:** Correlation of Constructs and AVEs.

Variable	AC	AR	SN	PN	PBC	Att.	Int.
AC	0.822						
AR	0.632	0.892					
PBC	0.593	0.565	0.838				
PN	0.648	0.595	0.638	0.871			
SN	0.475	0.396	0.526	0.457	0.817		
Attitude	0.462	0.410	0.441	0.428	0.394	0.839	
Intention	0.379	0.335	0.433	0.348	0.397	0.440	0.766

Notes: Square roots of the AVEs (average variance extracted) are on the diagonal, and correlations between constructs are on the off-diagonal; AC = Awareness of consequence of car-transport; AR = Ascription of responsibility of car-transport reduction; PN = Personal norm of car-transport reduction; SN = Subjective norm of car-transport reduction; PBC = Perceived behavioral control over car-transport reduction; Attitude = Attitude towards car-transport reduction; and Intention = Intention to reduce car-transport.

**Table 4:** Hypothesis Test Results.

**Paths**	**β (S.E.)**	***t***	**Results**
AC→PN	0.32 (0.04)	7.66 ***	H1a supported
AR→PN	0.20 (0.04)	5.39 ***	H2a supported
PN→Intention	0.12 (0.04)	2.80 **	H3 supported
Attitudes→Intention	0.30 (0.04)	7.00 ***	H4 supported
SN→PN	0.33 (0.04)	8.12 ***	H5 supported
PBC→Intention	0.23 (0.04)	5.72 ***	H6 supported
SN→Intention	0.04 (0.02)	2.57 *	H7a supported
**Mediation Effects**	**β (S.E.)**	**95% CI**	**Results**
AC→PN→Intention	0.11 (0.04)	[0.04, 0.19]	H1b supported
AR→PN→Intention	0.13 (0.04)	[0.06, 0.22]	H2b supported
SN→PN→Intention	0.08 (0.04)	[0.002, 0.38]	H7b supported

Notes: AC = Awareness of consequence of car-transport; AR = Ascription of responsibility of car-transport reduction; PN = Personal norm of car-transport reduction; SN = Subjective norm of car-transport reduction; PBC = Perceived behavioral control over car-transport reduction; Attitudes = Attitudes towards car-transport reduction; Intention = Intention to reduce car-transport. * *p* < 0.05; ** *p* < 0.01; *** *p* < 0.001.
